# Effect of temporary freezing on postmortem protein degradation patterns

**DOI:** 10.1007/s00414-023-03024-y

**Published:** 2023-06-03

**Authors:** Janine Geissenberger, S. Pittner, B. Ehrenfellner, L. Jakob, W. Stoiber, F. C. Monticelli, P. Steinbacher

**Affiliations:** 1 Department of Environment and Biodiversity, University of Salzburg, Hellbrunner Straße 34, 5020 Salzburg, Austria; 2 Department of Forensic Medicine and Forensic Psychiatry, University of Salzburg, Salzburg, Austria

**Keywords:** PMI estimation; Muscle protein degradation; Pig, Frozen, Porcine model, Freeze-storage

## Abstract

**Background:**

A precise determination of time since death plays a major role in forensic routine. Currently available techniques for estimating the postmortem interval (PMI) are restricted to specific time periods or cannot be applied for individual case-specific reasons. During recent years, it has been repeatedly demonstrated that Western blot analysis of postmortem muscle protein degradation can substantially contribute to overcome these limitations in cases with different background. Enabling to delimit time points at which certain marker proteins undergo distinct degradation events, the method has become a reasonable new tool for PMI delimitation under various forensic scenarios. However, additional research is yet required to improve our understanding of protein decomposition and how it is affected by intrinsic and extrinsic factors.

Since there are temperature limits for proteolysis, and investigators are confronted with frozen corpses, investigation of the effects of freezing and thawing on postmortem protein decomposition in the muscle tissue is an important objective to firmly establish the new method. It is also important because freezing is often the only practical means to intermittently preserve tissue samples from both true cases and animal model research.

**Methods:**

Sets of dismembered pig hind limbs, either freshly detached non-frozen, or thawed after 4 months of freeze-storage (*n* = 6 each), were left to decompose under controlled conditions at 30 °C for 7 days and 10 days, respectively. Samples of the M. biceps femoris were regularly collected at predefined time points. All samples were processed via SDS-PAGE and Western blotting to identify the degradation patterns of previously characterized muscle proteins.

**Results:**

Western blots show that the proteins degrade predictably over time in precise patterns that are largely unaffected by the freeze-and-thaw process. Investigated proteins showed complete degradation of the native protein band, partly giving rise to degradation products present in distinct time phases of the decomposition process.

**Conclusion:**

This study provides substantial new information from a porcine model to assess the degree of bias that freezing and thawing induces on postmortem degradation of skeletal muscle proteins. Results support that a freeze–thaw cycle with prolonged storage in frozen state has no significant impact on the decomposition behavior. This will help to equip the protein degradation–based method for PMI determination with a robust applicability in the normal forensic setting.

**Supplementary Information:**

The online version contains supplementary material available at 10.1007/s00414-023-03024-y.

## Introduction

A precise determination of time since death, also referred to as the postmortem interval (PMI), is a primary task in forensic routine work. The PMI is defined as the time elapsed since death, i.e., in forensic cases the time between the onset of death and the finding of a dead body [1]. In recent years, it has become increasingly evident that analysis of postmortem degradation patterns of muscle proteins is a promising new tool adding to the currently available methods for PMI estimation, especially because it expands the methodical scope into mid- and long-term PMI range. Utilizing established SDS-PAGE-based Western blot technology, this innovative approach has demonstrated that the degradation events of some muscle proteins reliably correlate with postmortal timespans in animal models and in humans [2–4]. Relevant information comes from both the degradation of the native protein itself and degradation-derived split products emerging at distinct time points after death.

However, earlier studies on the subject have also indicated that individual circumstances of death and several internal and environmental factors can influence the decomposition process, thus complicating PMI estimation. Resulting restrictions are well known from established methods of PMI determination, for instance, the cooling rate–based nomogram method, commonly applied in the early postmortal phase [5], and evaluation of colonization by arthropods (forensic entomology), mainly applicable in later postmortal phases [6–10], and also from analysis of volatile organic compound (VOC) species [11–13]. For all established and currently applied PMI estimation methods, it is clearly apparent that especially temperature is a fundamental influencing factor [5, 6, 14–16].

In order to ensure the precision and the applicability of the protein degradation–based method amongst a broad variety of forensic cases, recent research has already investigated the influence of some factors and individual traits, including sex, age, body mass, and temperature. It was found that there are significant correlations between postmortem protein degradation and age and body mass (as expressed by BMI), whereas sex did not exert measurable effects [17]. Also regarding influence from mass and volume, our own research was able to exclude that porcine postmortem protein degradation is significantly altered, depending on whether muscle samples are taken from dismembered limbs or from limbs still attached to the whole animal [18]. Expectedly, evidence was obtained that temperature acts without doubt as a major, if not as the most important factor on muscle protein degradation. Studies on animals and humans in forensic research [9, 19–21], as well as meat science studies [13], have shown that autolytic and decomposition dynamics are accelerated at higher temperatures but delayed under cool conditions.

A prominent gap in our knowledge of temperature effects on postmortem degradation of muscle proteins still exists in relation to the degree of bias by shorter or longer exposure or storage at temperatures below 0 °C. A freeze–thaw history is not uncommon in forensic practice. Frozen corpses are found outdoors under freezing conditions, and body parts of crime victims have been hidden in freezers [22, 23]. In such cases, it is often hard [24], if not impossible [9], to determine the time since death.

As with other information on the subject, some data on how a freeze–thaw treatment acts on muscle proteins have come from research on meat quality. Studies investigating aspects of meat quality including tenderness, water-holding capacity, and optimal meat storage conditions for different livestock species observed distinct changes in myofibrillar proteins and proteases [25–27]. When comparing muscle samples stored at temperatures above the freezing point with samples subjected to the “superchilling” conservation method (involving maintenance at − 0.5 °C to − 4 °C), researchers found that the degradation of some proteins was significantly slower under the latter regime [13]. Another study demonstrated that freeze–thaw treatment advances the degradation in some proteins (e.g., desmin) while leaving others unaffected (e.g., troponin T) [28].

Given this background, it seems evident that there is still need for research to clarify to which extent a freeze–thaw process influences the decomposition of muscle proteins employed in protein degradation–based PMI determination.

To address this task, this study uses a standardized protein degradation model that was implemented in previous work [18, 29]. We compare two sets of dismembered pig hind limbs (*n* = 6 each), one of which was left to degrade in a climate chamber at 30 °C immediately after slaughter, whereas the second set was freeze-stored at − 20 °C for 4 months before being subjected to degradation under the same conditions. During each experiment, samples were taken from the M. biceps femoris at regular intervals, and degradation patterns of selected proteins (vinculin, alpha tubulin, alpha actinin, glyceraldehyde-3-phosphate dehydrogenase (GAPDH)) were analyzed via SDS-PAGE followed by Western blotting. Proteins were selected according to their previously characterized differences in degradation rates. These degradation events occur either rather early (within 1 or 2 days) or in later postmortem phases (several days) and lie within the observed time course of the present study. Results are thought to improve our understanding of if and how a freeze–thaw process interferes with PMI determination, based on muscle protein degradation, thus adding to the robustness of this forensic methodology.

## Materials and methods

### Experimental design and sampling procedure

Six commercial sub-adult crossbreed pigs (German Large White × German Landrace, 4 months old, 50 ± 4 kg, 4 male, 2 female) were used for the experiment. To minimize possible bias from factory farming practices, experimental animals fed on quality feed from local production were obtained from controlled species-appropriate husbandry. Animals were killed in a certified slaughterhouse according to standard procedures by captive bolt stunning and subsequent exsanguination. Both hind limbs of each individual were separated by dissection with professional butcher cutlery immediately after death, giving a total of *n* = 12 limbs. A first set of samples from the M. biceps femoris (= 0 h reference samples) was taken from all hind limbs still in the slaughterhouse immediately after death, prior to transportation to the lab. For further processing, hind limbs were allocated to two treatment groups (*n* = 6) with different experimental setups.

The first group (referred to as “non-frozen”) was, without further delay, transferred to a climate chamber and incubated under constant conditions (temperature 30 ± 2 °C; humidity 50 ± 5% rH). Muscle samples were regularly collected at a total of 18 pre-defined time points after death: 0, 4, 8, 12, 16, 24, 32, 40, 48, 56, 64, 72, 80, 96, 112, 128, 144, 160 h postmortem (hpm).

**Fig. 1:**
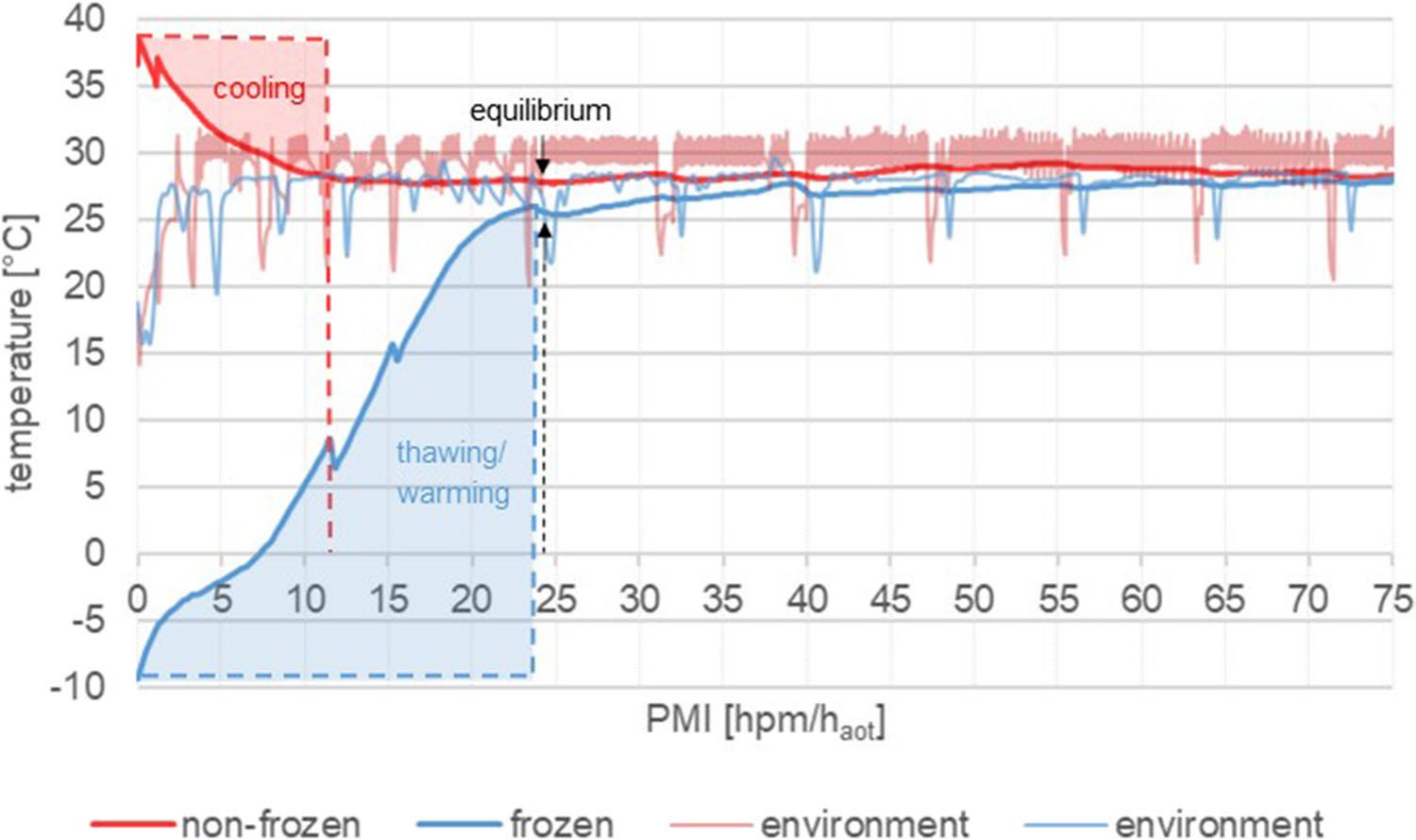
Temperature measurements throughout the first 75 h of the experiment until constant conditions were reached. Red lines indicate non-frozen rectal (*thick line*) and environmental (*thin line*) temperatures. Blue lines represent temperatures of frozen hind limbs. Temperature measurements of frozen limbs start with the onset of thawing/warming process (marked in transparent blue). Temperature data show that after approx. 12 h, the non-frozen hind limbs have cooled down to ambient temperature (marked in transparent red), whereas the frozen hind limb reached the equilibrium after approx. 24 h. After approx. 24 h, both treatment groups were at similar temperature conditions (Note: transient drops in environmental temperatures are due to opening of the climate chamber during sample collection.)

The second group of hind limbs (referred to as “pre-frozen”) was first subjected to long-time storage in a deep-freeze room at constant − 20 °C for 4 months and then transferred and stored in the + 30 °C climate chamber under the same conditions applied to the non-frozen limbs. Sampling also followed the time schedule of the non-frozen limbs, although with two modifications: (i) The pre-frozen limbs were sampled in still frozen condition, directly after the transfer to the climate chamber (for technical modifications, see below). This provided the 0*h_aot_ baseline samples (h_aot_ = hours after onset of thawing), containing all marker proteins at onset-of-thawing state. (ii) Since full adaptation to + 30 °C took approximately 24 h (cf. Fig. 1), sampling of pre-frozen limbs was prolonged for another day. This enabled us to take two additional samples (at 168 and 184 h_aot_, respectively) in order to ensure high comparability of the two experimental groups. Note: Sampling times of the pre-frozen group are expressed in hours after onset of thawing [h_aot_]. However, in the interests of simplicity, in the present work, h_aot_ is often treated as equivalent to hpm.

Sampling in both treatment groups was performed according to the following standardized procedure: An incision was made through the skin and the underlying fascial layer using a surgical scalpel, and muscle samples at a size of approx. 5 × 5 × 5 mm were excised from the M. biceps femoris at a depth of 2 cm within the belly of the muscle. A minimum distance of 2 cm was kept between successive sampling sites. Muscle samples were snap frozen and stored in liquid nitrogen until further processing. As the still-frozen condition of the limbs at 0*h_aot_ prevented scalpel incisions, these samples were obtained with the help of a power drill. After discarding skin and fatty tissue, borings of muscle tissue from a depth of 2 cm were collected and transferred to liquid nitrogen.

### Temperature measurements

During both experimental time courses (non-frozen and pre-frozen treatment groups), environmental conditions in the climate chamber, as well as temperature data inside of one hind limb, as measured by a puncture sensor, were documented throughout the entire sampling time (cf. Fig. 1).

Temperature measurements of frozen limbs start with the onset of thawing/warming process, after a 4-month storage at − 20 °C. Temperatures of non-frozen hind limbs were documented directly after death and after being placed within the climate chamber. Temperature data show that after approx. 12 h, the non-frozen hind limbs have cooled down to ambient temperature, whereas the frozen hind took approx. 24 h. After approx. 24 h, both treatment groups were at similar temperature conditions.

Notably, during the placement of the hind limbs inside the chamber and setting up all the data loggers for temperature measurements, the chamber was open and the temperature dropped. It took approximately one and a half hours for the chamber to adjust the temperature again. Transient drops in environmental temperatures occurred during sample collection due to opening of the climate chamber.

### Sample processing

Muscle samples were homogenized by cryogenic grinding and subsequent sonication with ultrasound (2 × 100 Ws/sample). A 10 × vol/wt RIPA buffer was used as lysis and extraction buffer, containing protease inhibitor cocktail (SIGMA) to prevent further protein degradation. Homogenized samples were centrifuged at 1^.^000 × *g* for 10 min, and supernatants transferred to separate tubes and stored at − 20 °C until further use. Total protein concentrations in the samples were measured using a Pierce BCA-Assay Kit (Thermo Fisher Scientific Inc.) and diluted to protein-specific values with double distilled water (30 µg for vinculin and alpha tubulin, 15 µg for alpha actinin, 10 µg for GAPDH).

### SDS-PAGE and Western blotting

SDS-PAGE was performed according to the protocol of Laemmli [30] with some adaptions. Electrophoresis was run on 5% stacking gels (acrylamide/N,N′-bismethylene acrylamide = 37.5:1, 0.1% SDS, 0.125% TEMED, 0.075% APS, 125 mM Tris HCl, pH 6.8) and 10% polyacrylamide resolving gels (acrylamide/N,N′-bismethylene acrylamide = 37.5:1, 0.1% SDS, 0.05% TEMED, 0.05% APS, 375 mM Tris HCl, pH 8.8). The running buffer contained 25 mM Tris pH 8.3, 195 mM glycine, 2 mM EDTA, and 0.1% SDS. Samples diluted to adequate total protein content (10–30 µg) were denatured at 90 °C for 5 min prior to insertion into the stacking gel wells (volume 20 µl). Electrophoresis was performed at a constant voltage of 150 V until the dye front reached the bottom of the resolving gel (duration approximately 2 h). Following electrophoresis, proteins were transferred from the gels onto polyvinylidene fluoride (PVDF) membranes in transfer buffer containing 192 mM glycine, 20% methanol, and 25 mM Tris pH 8.3. Electroblotting was run at a constant current of 250 mA for 75 min. Membranes were then stored at − 20 °C until further use. For Western blotting, membranes were blocked for 1 h in a blocking buffer containing PBST (137 mM NaCl, 10 mM Na_2_HPO_4_ anhydrous, 2.7 mM KCl, 1.8 mM KH_2_PO_4_, 0.05% Tween) including 1% bovine serum albumin (BSA; albumin bovine fraction V, pH 7.0) and then for 1 h incubated with the following primary antisera: mouse-clonal anti-vinculin (7F9, Santa Cruz Biotechnology, 1:1000), mouse monoclonal anti-α-actinin (H-2, Santa Cruz Biotechnology, 1:1000), mouse monoclonal anti-α-tubulin (12G10, DSHB, 1:500), and mouse monoclonal anti-GAPDH (6C5, Santa Cruz Biotechnology, 1:1500). HRP-conjugated polyclonal goat anti-mouse immunoglobulin (Dako, 1:10,000) was applied as secondary antibody. All antibodies applied were diluted in blocking buffer. After each antibody application, membranes were extensively washed and rinsed in PBST (3 × 10 min). HRP-mediated specific antibody binding was visualized with chemiluminescence substrate (Roti®-Lumin plus, Carl Roth) and photographed using an iBright CL1000 Imaging System (Thermo Fisher Scientific).

### Data interpretation and statistics

Band intensity of all proteins was measured using the gel analysis tool of the ImageJ software (v.1.48 NIH, National Institutes of Health, USA). Histograms of the tonal distribution of the images were plotted and the areas underneath the graphs were measured according to the program’s standard protocol. Band patterns of the 0-hpm samples were considered the native form of the protein and used as a control in both experimental series. All signals with ≥ 1% relative density (compared to the respective dominant control band) were considered a present protein band; all signals < 1% of the respective control band were considered background. This enabled binarization of the results and provided obtaining binary information of the absence (0) or presence (1) of proteins and their degradation products.

The abundance of bands per time point and the respective PMI [hpm] were then statistically analyzed and logistic regressions were calculated for all significant correlations of protein changes with a significance level above 0.95. This allows to predict the PMI at which the presence of a specific degradation product can be expected in a significant number of cases (confidence threshold = 95%) or at which time point the native protein (or splice variant) is completely degraded. The method also provides indication about when a change is more likely to have occurred than not (using *P* = 50% as a threshold). In addition, bivariate correlations between the chronology of protein degradation events and the PMI were calculated using Spearman’s rank correlation coefficient (Spearman’s *ρ*). For all proteins that gave a full set of data, i.e., those exhibiting distinct degradation events in all investigated hind limbs within the investigated time period of 160 hpm, an analysis of variance (ANOVA) was performed to evaluate possible differences between treatment groups. Since certain proteins showed no degradation in some of the hind limbs, their data had to be excluded from ANOVA evaluation since this specific data interpretation requires a “last time point an individual protein was present”. By simply using the last sampling point, it would most likely not represent the actual outcome because the protein band might be present long after the investigated time. Statistical analyses were performed using the SPSS Statistics 26 software (IBM, USA), MS Excel 2016, and RStudio (PBC).

## Results

### Morphological observations

**Fig. 2:**
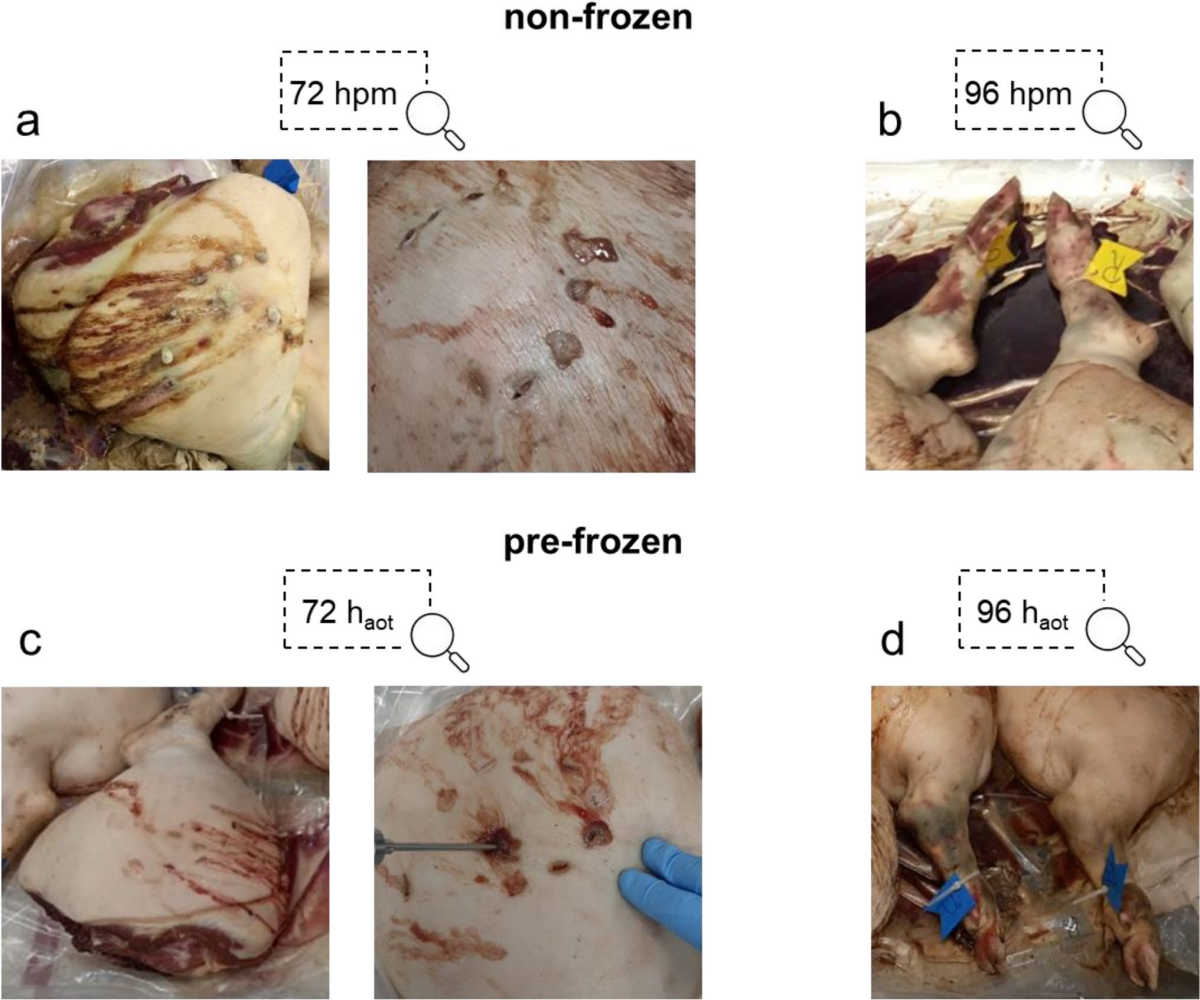
Morphological changes during the decomposition process. Detail pictures of non-frozen (**a**, **b**) and pre-frozen (**c**, **d**) hind limbs at 72 hpm/h_aot_ and 96 hpm/h_aot_ show extensive bloating and foam formation at incision sites from 72 hpm/h_aot_ onwards (**a**, **c**). In the course of the decomposition process, both frozen and non-frozen pig legs developed green(ish) discolorations, especially at the tarsal region from 96 hpm/h_aot_ onwards (**b**, **d**)

During the decomposition process, the hind limbs of both experimental groups (non-frozen vs. pre-frozen) exhibited similar characteristic morphological changes (cf. Fig. S1). Gas-driven bloating of the limb tissue mass and discharge of yellowish foam at incision sites were observed from 72 hpm onward (Fig. 2a, c) and could be detected until the end of the experiment in both groups. Discoloration of the skin, including black and greenish spots, was first detectable at 96 hpm, especially in the tarsal regions in both non-frozen (Fig. 2b) and pre-frozen hind limbs (Fig. 2d).

### Characteristics of postmortem protein degradation

Western blot results from all baseline samples — i.e., the 0-h samples taken immediately after slaughter from both groups and the 0*h_aot_ samples taken at onset of thawing from the pre-frozen group — showed no qualitative differences in protein degradation patterns (Fig. S2), apart from slight variances in band intensities of some proteins.

Data of the temperature-monitored pre-frozen hind limb show that it took approximately 7 h until they were fully thawed, followed by a steep further increase of temperature until adaption to the environmental temperature in the climate chamber (30 °C). Non-frozen hind limbs took about 12 h to adapt from physiological temperature (38/39 °C) at slaughter to climate chamber temperature. Thus, both treatment groups were at similar temperature conditions from approximately 24 h onwards. Temperature measurements were terminated after 75 hpm/h_aot_ after data showed constant conditions over a period of time. (Fig. 1).

In general, marker protein degradation in the two experimental groups followed similar qualitative patterns. Although minor temporal differences were observed in some proteins, statistical analysis showed no significant differences between pre-frozen and non-frozen hind limbs (Fig. 4, Table 1).

**Table 1:** Presence probability data of protein bands and analysis of variance (ANOVA) of non-frozen and pre-frozen hind limbs, including *P* = 50% values (taken indicative of whether a degradation event is more likely to occur than not). Data all together do not reveal any major differences between treatment groups

		Presence probability [hpm/h_aot_]	Analysis of variance (ANOVA)
		*P* = 50% ± CI (90%)	*p* value
Meta-vinculin	Non-frozen	45.3 ± 11.0	0.112
	Pre-frozen	38.2 ± 30.9	
Native vinculin	Non-frozen	79.7 ± 22.3	0.309
	Pre-frozen	81.3 ± 11.7	
Vinculin 90 kDA	Non-frozen	74.7 ± 43.8	0.512
	Pre-frozen	84.1 ± 26.7	
Native alpha tubulin	Non-frozen	102.4 ± 69.0	n/a
	Pre-frozen	108.2 ± 97.1	
Native alpha actinin	Non-frozen	135.2 ± 68.8	n/a
	Pre-frozen	116.6 ± 52.2	
Alpha actinin 60 kDa	Non-frozen	84.5 ± 69.8	0.059
	Pre-frozen	100.7 ± 65.0	
Native GAPDH	Non-frozen	154.3 ± 31.0	n/a
	Pre-frozen	137.2 ± 77.3	

**Fig. 3:**
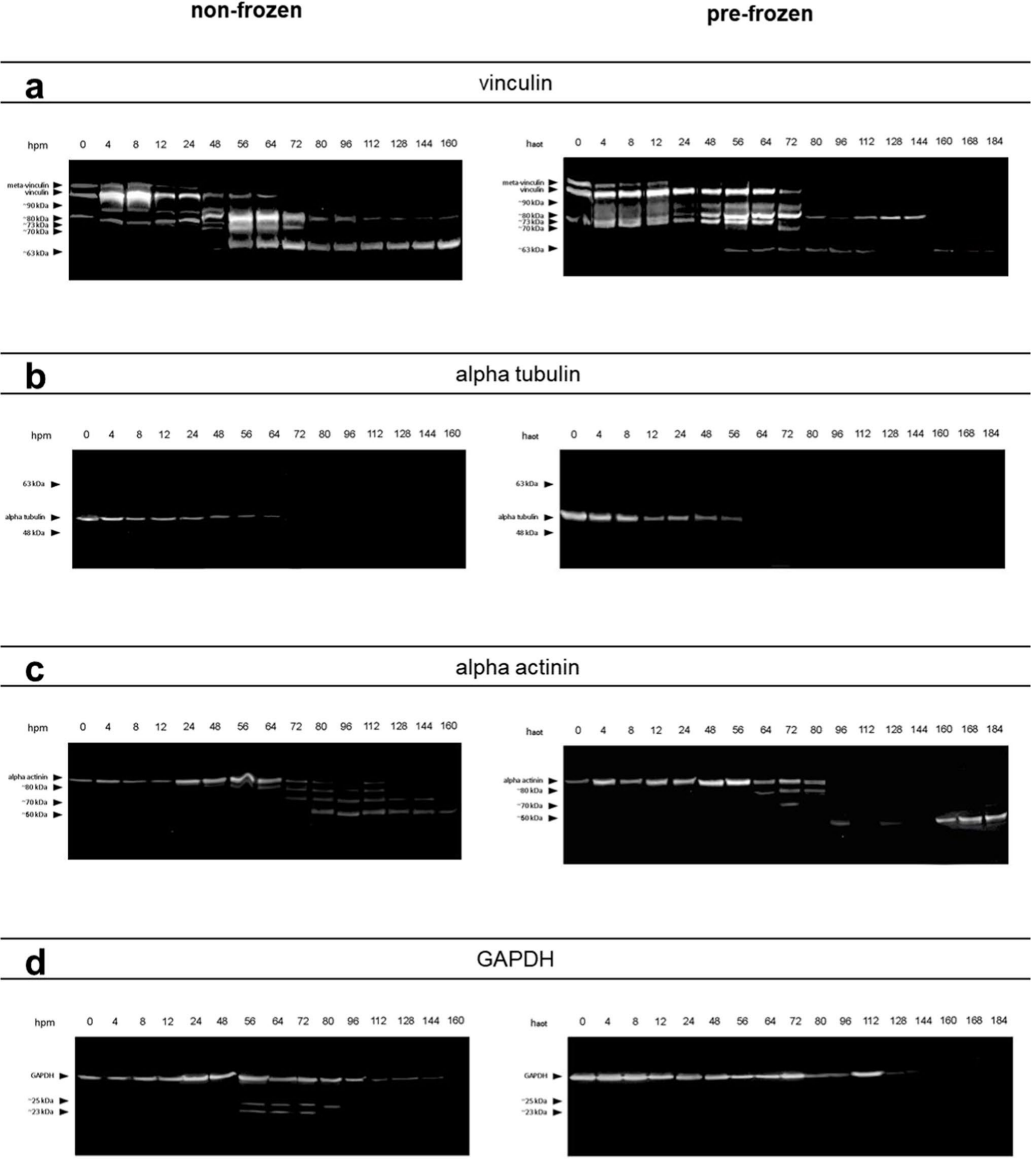
Representative Western blot results showing fading of native vinculin (**a**), alpha tubulin (**b**), alpha actinin (**c**), and GAPDH (**d**) bands in both non-frozen (left) and frozen hind limbs (right). The degradation is accompanied by the occurrence of degradation products at specific time points (**a**, **c**, **d**)

**Fig. 4:**
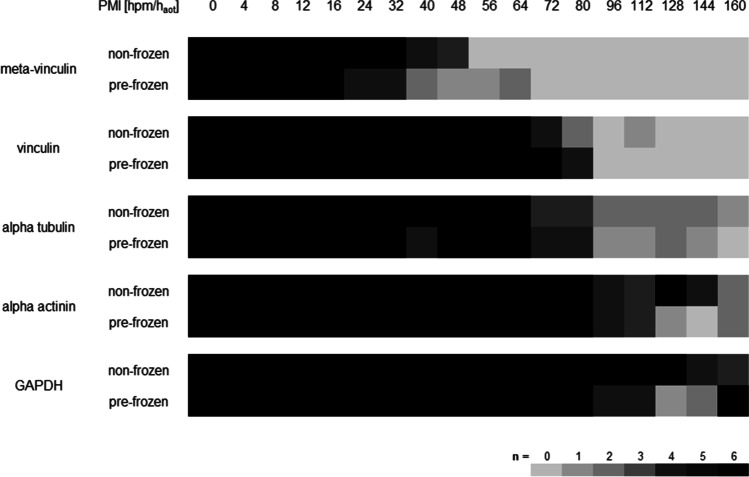
Heatmap analysis of investigated proteins showing the abundance of native protein bands of vinculin, alpha tubulin, alpha actinin, and GAPDH, and of the splice variant meta-vinculin. Note the decrease in all proteins over the investigated time course in both treatment groups (non-frozen and pre-frozen)

**Fig. 5:**
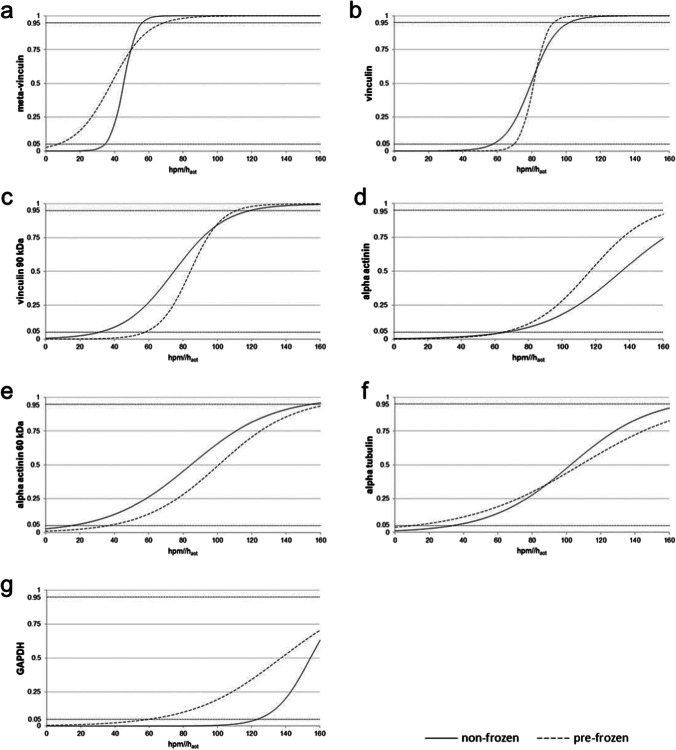
Probability of presence of significantly PMI-correlated proteins and their degradation products over the investigated time period as depicted by logistic regressions. Regression curves (non-frozen:* solid line*; frozen:* dashed line*) of meta-vinculin (**a**), vinculin (**b**), vinculin 90 kDa (**c**), alpha actinin (**d**), alpha actinin 60 kDa (**e**), alpha tubulin (**f**) and GAPDH (**g**) are plotted within the PMI range from 0 to 160 hpm/h_aot_. The probability of all degradation events increases with increasing PMI. There is no specific trend regarding the steepness of the curves in any of the treatment groups. Some proteins of the pre-frozen group exceed the 95% confidence limit in advance of their non-frozen counterparts, and vice versa

In detail, vinculin presented a complete degradation within the time of the experiment, concerning both the 117-kDa native protein band and an additional band at approx. 135 kDa representing the splice variant meta-vinculin [31, 32] (Figs. 3a and 4). In both experimental groups, the presence of the native vinculin band (non-frozen: *ρ* = 0.867, *p* < 0.01; pre-frozen: *ρ* = 0.890, *p* < 0.01), and even more so the presence of the meta-vinculin band (non-frozen: *ρ* = 0.867, *p* < 0.01; pre-frozen: *ρ* = 0.890, *p* < 0.01), significantly correlate with the PMI (Tab. S1). Regressions revealed that the native protein band was significantly present until 92.9 hpm in pre-frozen hind limbs and until 102.1 hpm in non-frozen hind limbs (> 95% likelihood). The splice variant was present until 69.1 hpm in frozen legs and until 56.4 hpm in non-frozen legs (Fig. 5a, b; Table 1). In addition, analysis of variance (ANOVA) revealed that there was no significant difference (*p* > 0.05) in the degradation behavior between frozen and non-frozen hind limbs for both vinculin and its splice variant meta-vinculin (Table 1).

At distinct times in the progressing degradation, vinculin split products became detectable (Fig. 3a). In addition to the native band and the meta-vinculin band, a protein band of approx. 90 kDa appeared throughout the early pm period, again significantly correlated with the PMI (non-frozen: *ρ* = 0.874, *p* < 0.01; pre-frozen: *ρ* = 0.894, *p* < 0.01) (Tab. S1). Logistic regressions showed that this protein band was detectable in early postmortem stages until 118.5 hpm in non-frozen hind limbs and until 110.6 hpm in frozen hind limbs with a likelihood of > 95% (Fig. 5c; Table 1). Furthermore, *P* = 50% values of the 90 kDa vinculin band was statistically reached at 74.7 hpm in non-frozen hind limbs and at 84.1 hpm in pre-frozen limbs (Table 1) which indicates the time points when the occurrence/presence of this split product is more likely than its absence. Nevertheless, a comparison between treatment groups (non-frozen versus pre-frozen) via ANOVA again showed no significant difference (*p* > 0.05) between treatment groups (Table 1).

More varied circumstances characterize a set of further vinculin split products (Fig. 3a). All investigated samples exhibited bands at approximately 80 kDa, 76 kDa, 70 kDa, and 63 kDa, which were also identified as vinculin degradation products. Their presence was somewhat more varied, both along the time axis and between experimental groups. Irrespective of the experimental group, all these degradation products (except 63-kDa vinculin) showed no significant correlation between their presence and the PMI.

Alpha tubulin showed a single native protein band at about 49 kDa which remained stable until it fully degraded in the intermediate phase of the experiment (from approx. 64 hpm onwards) without giving rise to any split products (Figs. 3b and 4). This complete degradation occurred in all examined limbs except for one of the non-frozen limbs in which the native alpha tubulin band remained stable over the entire period of observation (thus analysis of variance was invalid). Logistic regressions revealed similar *P* = 50% values for both treatment groups of 102.4 hpm for non-frozen and 108.2 hpm for pre-frozen hind limbs. Both *P* = 95% values exceeded the investigated time period of 160 hpm (Fig. 5f; Table 1). However, there is a highly significant correlation between the presence of native alpha tubulin and the PMI in both frozen and non-frozen limbs (*ρ* = 0.795, *p* < 0.01 and *ρ* = 0.905, *p* < 0.01, respectively) (Tab. S1).

Alpha actinin presented a relatively stable native band at a molecular weight of about 100 kDa, the intensity of which faded with increasing PMI in 4 of 6 non-frozen limbs and in all pre-frozen limbs until complete disappearance (Figs. 3c and 4). In two of the non-frozen legs, this protein remained stable over the investigated time, preventing ANOVA between treatment groups. All investigated limbs exhibited several degradation products of alpha actinin, with molecular weights at approx. 80 kDa, 70 kDa, and 60 kDa in the middle and late phases of the experiment (from 48 hpm onwards) (Fig. 3c). Notably, these degradation products appeared later and persisted longer in pre-frozen limbs than in non-frozen limbs. There is a highly significant correlation between the presence of native alpha actinin and the PMI in both frozen and non-frozen limbs (*ρ* = 0.873, *p* < 0.01 and *ρ* = 0.833, *p* < 0.01 respectively). Except for the 80-kDa band, also the presence of alpha actinin degradation products correlates strongly with the PMI (Tab. S1). Logistic regressions showed that *P* = 50% values of native alpha actinin were reached at 135.2 hpm for non-frozen, and at 116.6 hpm for pre-frozen hind limbs (Fig. 5d; Table 1). The alpha actinin degradation product of approx. 60 kDa showed a presence probability of 50% at 84.5 hpm in non-frozen, and at 100.7 hpm in pre-frozen pig legs (Fig. 5e; Table 1). In addition, ANOVA variance analysis showed no significant difference (*p* > 0.05) between non-frozen and pre-frozen hind limbs (Table 1).

GAPDH displayed a stable native protein band at approximately 35 kDa in all limbs until it degraded in the later phase of the experiment at about 144 hpm (Figs. 3d and 4). Statistical analysis revealed *P* = 50% values of 154.3 hpm in non-frozen hind limbs, and of 137.2 hpm in pre-frozen legs (Fig. 5g; Table 1). Additionally, two degradation products of GADPH at molecular weights of 25 kDa and 23 kDa were observed, although with varying incidence and timing between the experimental groups. These products were detectable in 5 of the 6 non-frozen limbs (in the time from 4 to 112 hpm), but in only 2 of the 6 pre-frozen limbs (irregularly during the first 72 h), the remaining limbs each containing none of them at all. Neither native GAPDH nor its degradation products showed a significant correlation of presence with PMI, except native GAPDH of the non-frozen limbs (*ρ* = 0.858, *p* < 0.01) (Tab. S1).

## Discussion

In this study, we employed a previously implemented protein degradation–based method of PMI determination [4, 18, 33] in a pig model to examine the effects of a freeze and thaw history of the analyzed tissue. Degradation patterns of pre-identified muscle proteins from porcine hind limbs subjected to controlled decay either immediately after slaughter, or after 4 months of freeze storage at − 20 °C were monitored and compared. Experimental animals from species-appropriate husbandry, a dense sampling scheme also taking into account the 24-h thawing and warming phase of the freeze-stored muscle, and target proteins already known to be appropriate for the method provide for predictable and reproducible results in the porcine test system used. We provide new information on the degradation behavior of mammalian muscle proteins in two relevant directions: First and foremost, the results of this experiment provide — to our knowledge for the first time — clear indication that transient freezing of muscle tissue does not significantly confound subsequent degradation proteins under warm (+ 30 °C) conditions, as compared to instant degradation of fresh non-frozen controls. Neither the patterns of gross-morphological changes (Fig. 2) nor the degradation behavior and kinetics of the proteins chosen for the experiment (vinculin, alpha tubulin, alpha actinin, GADPH) differed substantially between the two experimental settings (Figs. 3, 4, and 5; Table 1; Fig. S2). Statistical analysis showed no specific trend whether protein changes are more likely delayed in pre-frozen hind limbs, or in the non-frozen control limbs (Fig. 5; Table 1). The proteins alpha tubulin and alpha actinin remained stable in some of the non-frozen samples but not the pre-frozen samples. Whether the stability of these proteins in individual legs is a trend towards delayed or accelerated degradation processes in pre-frozen tissue can only be speculated. However, we did not observe similar outcome in other proteins. Furthermore, results demonstrate an evident correlation between protein changes and PMI, regardless of the treatment (Tab. S1). When applicable, an analysis of variance (ANOVA: single factor) was performed and revealed no significant difference between treatment groups (non-frozen versus pre-frozen) in any of the tested markers. Particularly, the protein changes of meta-vinculin, native vinculin, the 90-kDa vinculin degradation product, and the 60-kDa alpha actinin degradation product occurred at specific time points and showed no significant difference (*p* > 0.05), whether hind limbs were pre-frozen or not (Table 1). These findings contribute to increase the validity and reliability of the protein degradation–based method of PMI determination in forensic research and routine casework, if only because it is often necessary to freeze tissue samples for analysis at a later time. The present work clearly indicates that postmortem protein decomposition is arrested in frozen state. After thawing, however, the protein decomposition is continued, regardless of and uninfluenced by the earlier freezing process. This is especially important for forensic cases found outdoor during or right after freezing seasons, and when bodies are hidden in a freezer after a committed crime.

In addition, but not less importantly, the present results demonstrate for the first time that skeletal muscle is also a valid substrate for protein degradation–based PMI determination in cases with a freeze–thaw history. By showing that the degradation behavior of the selected proteins is robust against freezing, these results contribute to clarify an as yet largely undecided issue. Previous work targeting a variety of proteins from both muscle and inner organs had delivered a mixture of for and against a freeze–thaw influence, also with differences between organ-specific isotypes. This is already best exemplified by meat quality studies, some of which are principally in line with the present results [34, 35] while others, investigating a set of sarcoplasmic and myofibrillar proteins rather indicate that protein degradation is altered after a freeze–thaw cycle [11–13]. An ambiguous picture also resulted from comparison of protein isotypes from porcine muscle and brain. The cerebral isotypes including those of alpha tubulin and GADPH behaved in largely similar fashion as the muscle isotypes, regardless of whether deriving from pre-frozen or non-frozen brain tissue. Time courses of change, however, differed considerably when compared to those of the proteins’ muscle isotypes, cerebral alpha tubulin being fully degraded already after 10 hpm whereas cerebral GAPDH proved stable over the entire investigated time of approximately 50 hpm [36]. A similarly heterogeneous picture, although depicting an exceptional position of muscle, has been drawn by studies of thawing effects at the metabolomics level comparing multi-tissue samples (plasma, gut, kidney, liver, pancreas), also including muscle. Compared to muscle, thawed non-muscle samples were found to be characterized by higher levels of amino acids and other metabolites, likely the result of enhanced protein degradation, and a higher susceptibility to oxidation [37, 38]. In line with this, recent work from our own lab had shown muscle proteins including vinculin, alpha tubulin, and GAPDH exhibit similar degradation patterns irrespective of whether analyzed fresh or after a brief freeze–thaw cycle of 1 week [29], thus supporting target isotype choice for the present work.

Second, and not directly relating to the freeze–thaw context, the present results also expand upon the present knowledge on the general relationship between muscle protein degradation and temperature. Research to date has generally confirmed that the postmortal progression of human protein degradation in the thermal range of 4–37 °C (i.e., at the temperatures usually prevailing in the warm temperate zone) correlates tightly with ambient temperature [25–27, 39–41]. However, information on protein behavior within this range is still unequally distributed, evidence from the ranges’ upper segment (corresponding to warmer/hot climates) being largely missing. The present work is the first to provide robust data on how four proteins relevant to PMI determination degrade in an environment at + 30 °C, at least one step further toward the full set of reliable temperature correcting factors, which is crucial to provide the protein degradation–based method of PMI determination with a worldwide scope of application.

## Conclusion

Degradation patterns of muscle proteins relevant to PMI determination in a porcine model system are not critically influenced by whether the analyzed tissue passes through a (4 months) period of freezer storage at stable temperature. The findings give a promising perspective for a broad applicability of protein degradation–based PMI determination, while some caveats remain: The present experiment was based on a single freeze–thaw cycle only. This certainly mimics the conditions encountered by lab freezer–stored tissue, but does not fully apply to the erratically recurring freeze–thaw events to which corpses may subjected under real outdoor conditions. Verification in both thermally more fluctuating animal and human cadaveric models, importantly including field work, must follow to validate the present results.

### Supplementary Information

Below is the link to the electronic supplementary material.

Fig. S1 Morphological changes during the decomposition process of non-frozen (a) and pre-frozen (b) hind limbs over a time period of 160 hpm/h_aot_. Both experimental groups show similar changes over the investigated time and at certain time points. (PDF 459 kb)

Fig. S2 Representative Western Blots of vinculin, GAPDH, alpha actinin and alpha tubulin, depicting protein bands of non-frozen hind limbs at 0 hpm, and of hind limbs intended for freezing before freezing (pre freeze) and at the onset of thawing (post freeze). Results show no qualitative differences between the freeze-thawed samples and their pre-freeze references. Note that post-freeze samples exhibit no signs of degradation apart from slight fading of some of the protein bands (i.g. alpha tubulin). (PNG 93 kb)

High resolution image (TIF 233 kb)

Tab. S1 Bivariate correlations between the chronology of protein degradation events and the PMI calculated by using *Spearman’s rank correlation coefficient* (Spearman’s ρ and according *p* value). All native proteins (except for GAPDH of non-frozen hind limbs) and their specific degradation products show significant correlation (Spearman’s ρ ≥ 0.75) between protein changes and PMI. (JPG 122 kb)

## Data Availability

All data generated or analyzed during this study are included in the published article.

## Abbreviations

*DSHB*: Developmental Studies Hybridoma Bank
*GAPDH*: Glyceraldehyde-3-phosphate dehydrogenase
*haf*: Hours after freezing
*hpm*: Hours postmortem
*PMI*: Postmortem interval
