# Supplementary figures and images for: Effect of temporary freezing on postmortem protein degradation patterns
Source: Int J Legal Med. 2023 Jun 3;137(6):1803–14. doi: 10.1007/s00414-023-03024-y (PMC10567868; doi:10.1007/s00414-023-03024-y)

**non-frozen**

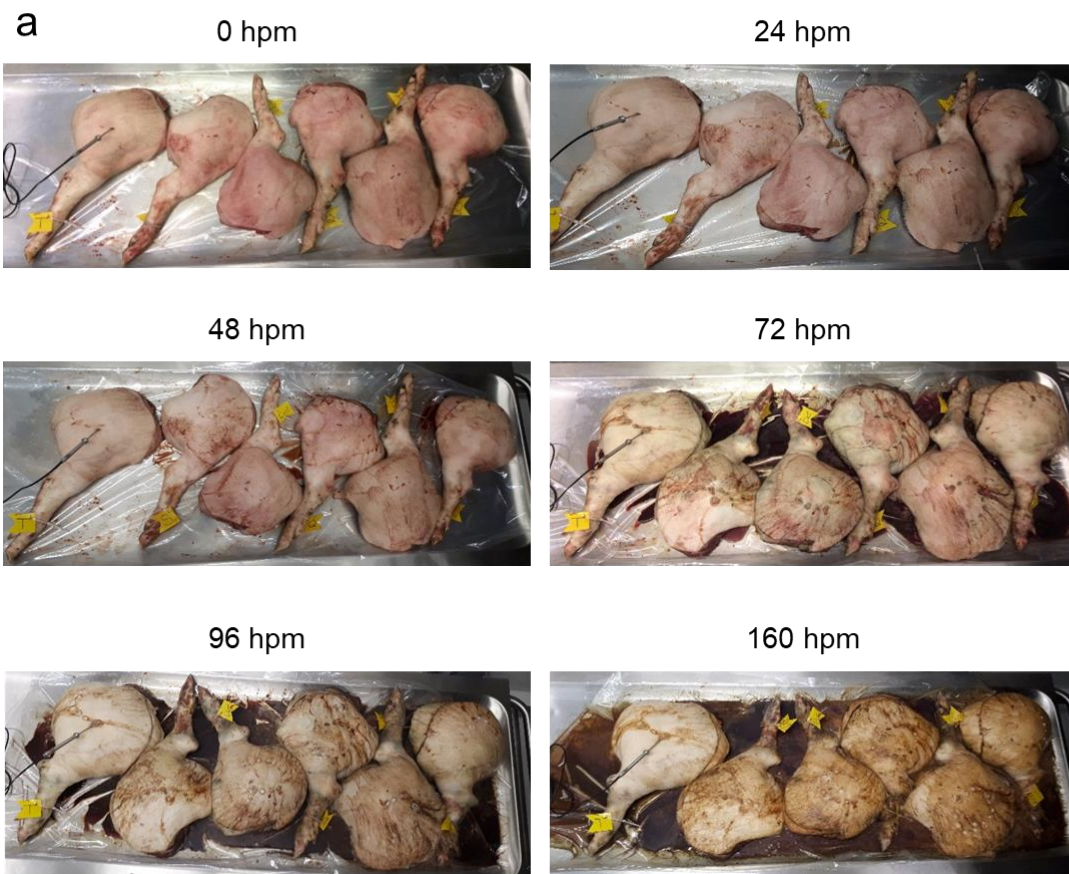

**pre-frozen**

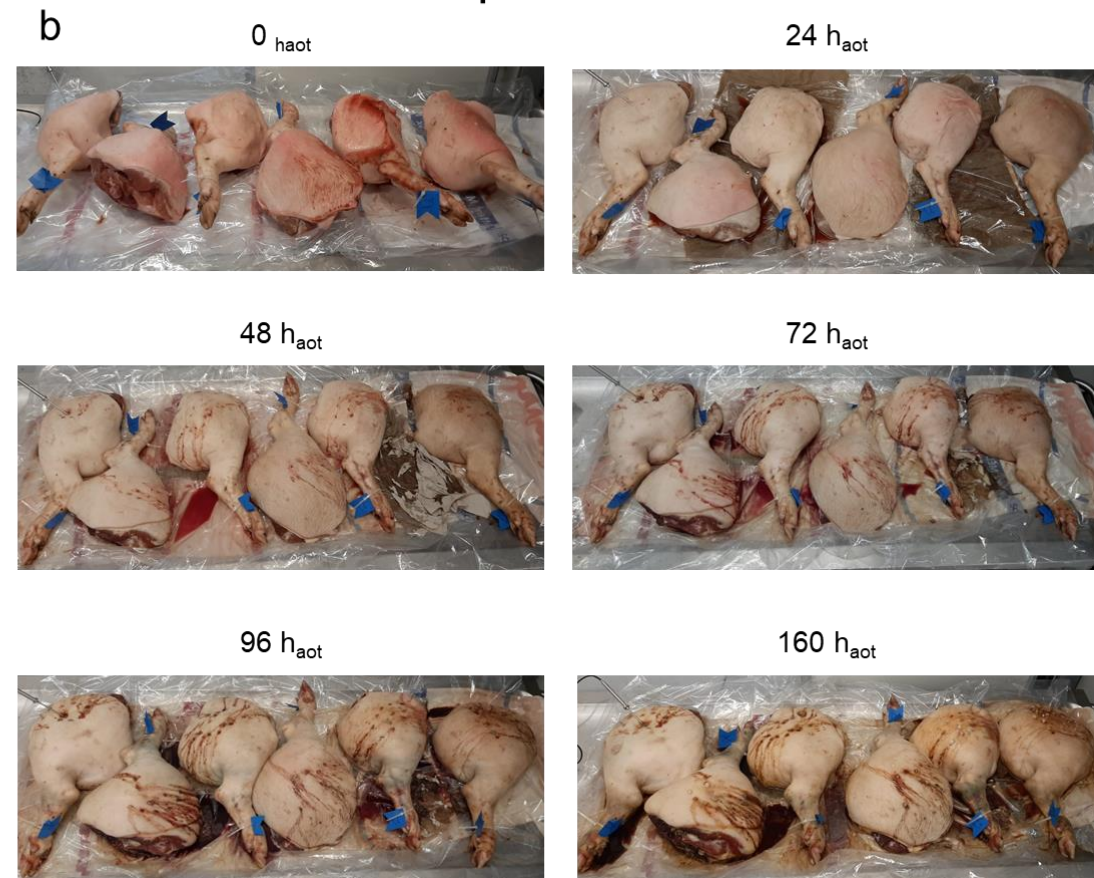

Supplement: Supplementary file 1 — Morphological changes during the decomposition process of non-frozen (a) and pre-frozen (b) hind limbs over a time period of 160 hpm/haot. Both experimental groups show similar changes over the investigated time and at certain time points. (PDF 459 kb) [file 414_2023_3024_MOESM1_ESM.pdf]

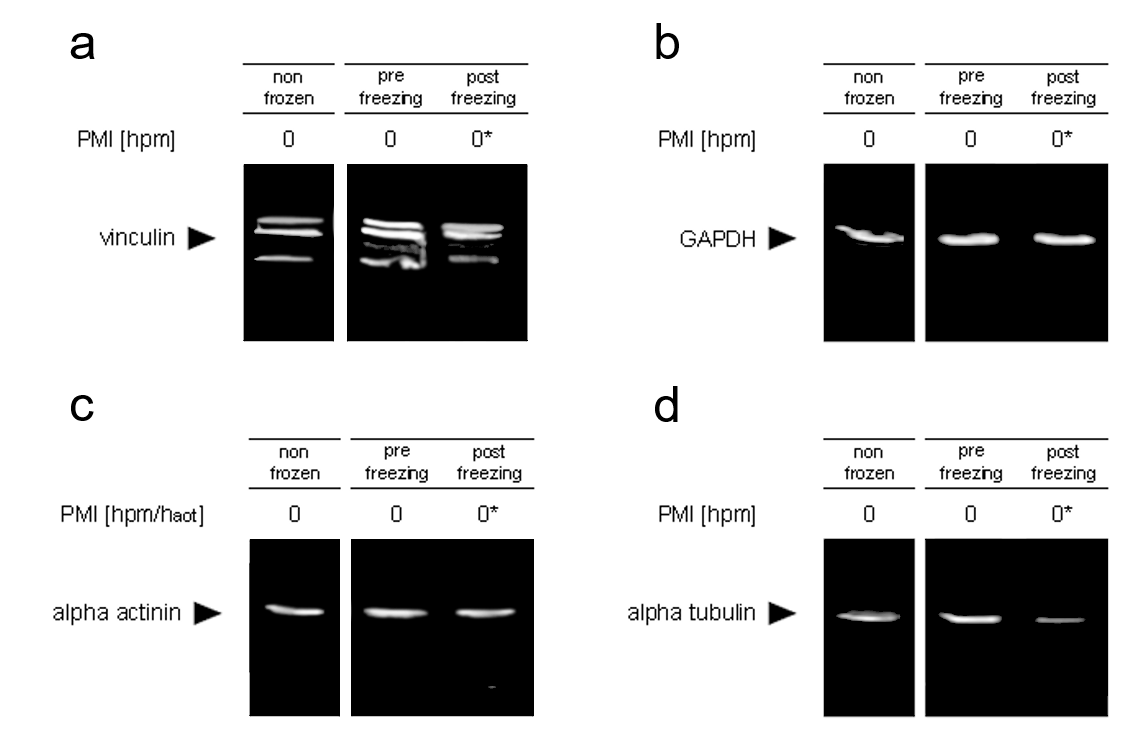

Supplement: Supplementary file 2 — Representative Western Blots of vinculin, GAPDH, alpha actinin and alpha tubulin, depicting protein bands of non-frozen hind limbs at 0 hpm, and of hind limbs intended for freezing before freezing (pre freeze) and at the onset of thawing (post freeze). Results show no qualitative differences between the freeze-thawed samples and their pre-freeze references. Note that post-freeze samples exhibit no signs of degradation apart from slight fading of some of the protein bands (i.g. alpha tubulin). (PNG 93 kb) [file 414_2023_3024_Fig6_ESM.png]

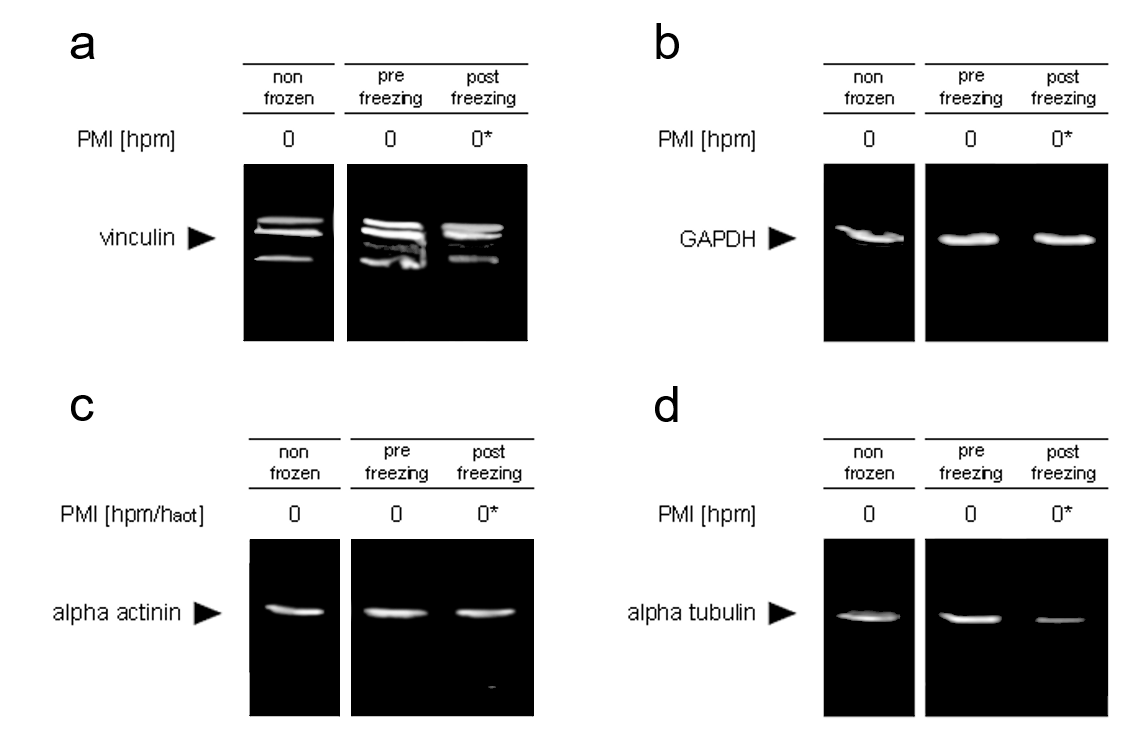

Supplement: Supplementary file 3 — High resolution image (TIF 233 kb) [file 414_2023_3024_MOESM2_ESM.tif]

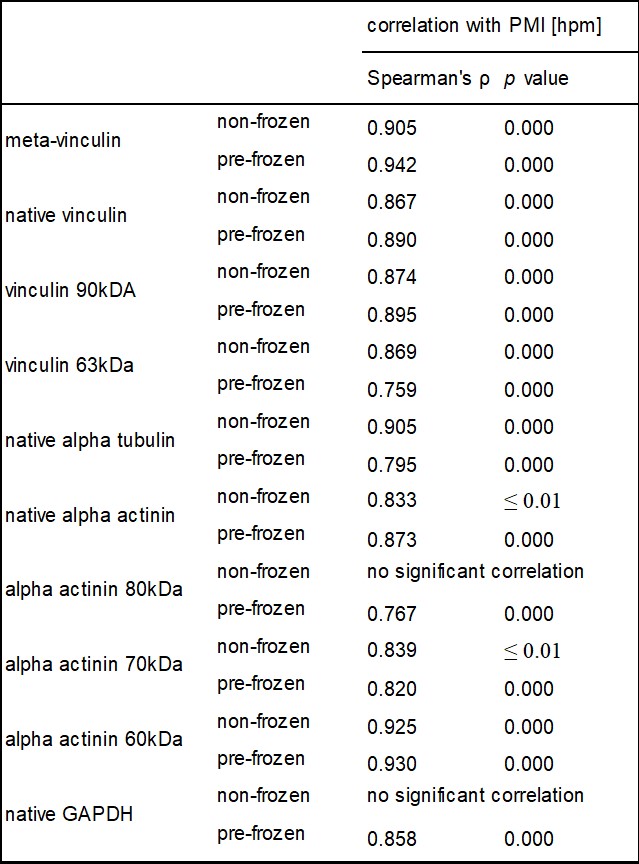

Supplement: Supplementary file 4 — Bivariate correlations between the chronology of protein degradation events and the PMI calculated by using Spearman’s rank correlation coefficient (Spearman’s ρ and according p value). All native proteins (except for GAPDH of non-frozen hind limbs) and their specific degradation products show significant correlation (Spearman’s ρ ≥ 0.75) between protein changes and PMI. (JPG 122 kb) [file 414_2023_3024_MOESM3_ESM.jpg]
